# Niraparib exhibits a synergistic anti-tumor effect with PD-L1 blockade by inducing an immune response in ovarian cancer

**DOI:** 10.1186/s12967-021-03073-0

**Published:** 2021-10-07

**Authors:** Jinyu Meng, Jin Peng, Jie Feng, Jochen Maurer, Xiao Li, Yan Li, Shu Yao, Ran Chu, Xiyu Pan, Junting Li, Ting Zhang, Lu Liu, Qing Zhang, Zeng Yuan, Hualei Bu, Kun Song, Beihua Kong

**Affiliations:** 1 Department of Obstetrics and Gynecology, Qilu Hospital, Cheeloo College of Medicine, Shandong University, 107 Wenhua Xi Road, Jinan, Shandong China; 2 Gynecology Oncology Key Laboratory, Qilu Hospital, Shandong University, Jinan, Shandong China; 3 Department of Obstetrics and Gynecology, University Hospital Aachen (UKA), 52074 Aachen, Germany; 4 School of Medicine, Cheeloo College of Medicine, Shandong University, Jinan, Shandong China; 5 Department of Obstetrics and Gynecology, The Sixth Hospital of Beijing, Beijing, China

**Keywords:** Ovarian cancer, Niraparib, PARP inhibitors, PD-L1, cGAS/STING pathway

## Abstract

**Background:**

Immune checkpoint blockades (ICBs) therapy showed limited efficacy in ovarian cancer management. Increasing evidence indicated that conventional and targeted therapies could affect tumor-associated immune responses and increase the effectiveness of immunotherapy. However, the effects of Niraparib, one of the poly (ADP) ribose polymerase (PARP) inhibitors, on the immune response remains unclear. Delineating the crosstalk between cytotoxic anticancer agents and cancer-associated immunity may lead to more efficient combinatorial strategies.

**Methods:**

Programmed death ligand 1 (PD-L1) expression in human ovarian cancer cells after PARP inhibitors treatment was examined by western blotting (WB) and flow cytometry. The expression of poly ADP-ribose polymerase (PARP1), PD-L1, and CD8 in human ovarian cancer tissues was detected by immunohistochemistry(IHC). The effect of Niraparib and PD-L1 blockade in ovarian cancer progression was investigated in vivo. The changes of immune cells and cytokines in vitro and in vivo were detected by flow cytometry and enzyme-linked immunosorbent assay (ELISA). Changes of cGAS/STING signal pathway after Niraparib treatment were determined by WB, ELISA.

**Results:**

Niraparib upregulated membrane PD-L1 and total PD-L1 expression in ovarian cancer cells and had a synergistic effect with PD-L1 blockade in vivo. In clinical patient samples, Niraparib augmented cytotoxic CD8^+^T cell proportion and function. In vivo and vitro, Niraparib can also increase the proportion of T cells and combined with PD-L1 blockade could further enhance the effect. Besides, Niraparib activated the cGAS-STING pathway, increasing the levels of cytokines such as CCL5 and CXCL10, which played a vital role in augmenting the infiltration and activation of cytotoxic T cells.

**Conclusions:**

Niraparib could modulate the immune response via the activation of the cGAS/STING pathway, and combination with PD-L1 blockade could further enhance the effect. These results provide a sound theoretical basis for clinical treatment.

**Supplementary Information:**

The online version contains supplementary material available at 10.1186/s12967-021-03073-0.

## Background

Ovarian cancer is the most lethal gynecological malignancy and the 5-year survival rate is below 48% [1], which can be attributed to its insidious nature, difficulties in early diagnosis, and chemotherapy resistance [2, 3]. In the United States, about 21,750 cases were confirmed and there were 13,940 deaths in 2020 [4].^.^Therefore, novel or combined strategies for ovarian cancer other than conventional chemotherapy are urgently needed [5].

Drugs targeting the DNA damage response (DDR) pathway such as Olaparib and Niraparib have been utilized for ovarian cancer therapy. Olaparib was first approved for the treatment of germline *BRCA*-mutated, platinum-sensitive, recurrent, and high-grade serous ovarian cancer [6]. Niraparib was recently approved by the United States Food and Drug Administration (FDA) for treating patients with recurrent, platinum-sensitive ovarian cancer after at least two previous chemotherapy treatments [7]. Despite the profound and sustained anti-tumor responses observed in treating ovarian cancer patients, resistance to PARP inhibitors has emerged in some cases [8]. To overcome this resistance, it is necessary to explore combinations with other agents, such as immunotherapies.

Although immune checkpoint inhibitors are promising, the response to these agents is limited and increasing evidence has shown primary and adaptive resistance [9]. It has been shown that responses to these agents are related to the mutagenic burden, such as in tumors with *BRCA* and other homologous recombination (HR) proteins deficiencies [10]. Moreover, PARP inhibitors have been reported to manifest synergistic effects with programmed cell death 1 ligand 1 (PD-L1) blockade in pre-clinical models and clinical trials [11]. However, the interactions between PARP inhibitors and the immune system as well as the underlying molecular mechanisms, remain unknown.

In our study, we investigated the dynamic changes and responses of immune cells and cytokines during Niraparib treatment. Our results showed that Niraparib could upregulate PD-L1 expression, but did not impair the function of immune cells, especially CD8^+^T cells. Interestingly, Niraparib enhanced the proportion and function of T cells via activation of the innate immune response pathway. Combined PD-L1 blockade could further promote the recruitment and activation of T cells. Our findings demonstrated that Niraparib exhibited a synergistic effect with immune checkpoint blockade (ICBs) in ovarian cancer therapy, which could shed light on ovarian cancer therapeutic management.

## Methods and materials

### Cell culture

Human epithelial ovarian cancer cell line UWB1.289 (BRCA1-null) was purchased from the American Type Culture Collection (ATCC). The SKOV3 and ID8 (murine ovarian carcinoma) cells were obtained from the Chinese Academy of Sciences (Shanghai, China). The SKOV3, ID8, and UWB1.289 cells were cultured in RPMI 1640 medium (GIBCO) with 10% fetal bovine serum (FBS), 100 U/ml penicillin, and 100 μg/ml streptomycin. All cells were maintained at 37 °C with 5% CO_2_ in a humidified incubator. PARP1 siRNA1, siRNA2, siRNA3 (siPARP1, Table 1) were purchased from GenePharma (Jiangsu, China). SKOV3 and UWB1.289 cells were transfected with siRNAs using Lipofectamine 2000 (Invitrogen, CA) according to the manufacture’s instructions, PARP1 and PD-L1 protein levels were determined by western blotting and flow cytometry.

**Table 1:** The sequence of PARP1 siRNA

No	Sequence	Sequence
PARP1-homo-2003	GAGCACUUCAUGAAAUUAUTT	AUAAUUUCAUGAAGUGCUCTT
PARP1-homo-1706	GAGGAAGGUAUCAACAAAUTT	AUUUGUUGAUACCUUCCUCTT
PARP1-homo-2699	GCGAAUGCCAGCGUUACAATT	UUGUAACGCUGGCAUUCGCTT

### Chemical inhibitors and antibodies

Olaparib (PARP inhibitor) was purchased from Selleck (TX, USA). Niraparib was kindly provided by ZaiLab (Shanghai, China). Niraparib was dissolved in dimethyl sulfoxide (DMSO) at a 10 mM concentration for the stock solution and diluted with culture medium to obtain a final DMSO concentration of no more than 0.1%. The antibody anti-human PD-L1 (SHR-1316) was kindly provided by Hengrui (Jiangsu, China). The blocking antibody anti-mouse PD-L1 (Rabbit mAb#10F.9G2) was purchased from Biolegend (San Diego, CA, USA). STING inhibitor (H-151) was purchased from Selleck (TX, USA).

### Patients and tissue samples

Seventy-two high-grade serous ovarian cancer (HGSOC) formalin-fixed, paraffin-embedded (FTPE) samples were collected from the pathology department and the clinical data were collected from the follow-up center of Qilu Hospital of Shandong University. The study was approved by the Ethics Committee of Qilu Hospital of Shandong University (Approval number: KYLL-2020-138).

### Syngeneic model

The previously cultured ID8 cells were resuspended in 100 μl of phosphate-buffered saline (PBS) at a volume of 1 × 10^7^ cells and injected subcutaneously into the right flank of female C57BL/6 mice aged 4–6 weeks were from Vitalriver (Beijing, China). After 2 weeks, the mice were randomized to four groups (n = 5 per group): control group, Niraparib (25 mg/kg) group, PD-L1 blockade group (10 mg/kg), and Niraparib (25 mg/kg) with PD-L1 (10 mg/kg) blockade group. Treatment was started when the tumors formed after two weeks of injection. A dose of 25 mg/kg Niraparib was administered to the mice orally 4 times per week, and PD-L1 blockade was injected intraperitoneally twice a week. Weight and tumor volume were measured every three days after treatment. The tumor volume was calculated as follows: V = a*b^2^/2 (V = volume, a = largest diameter of tumor, b = smallest diameter of tumor). The mice were sacrificed 8 weeks after the injection, and tumors were separated and weighed. Part of the tumor was used for the WB assay, and the rest was fixed with 4% paraformaldehyde for immunohistochemistry. The animal experiments were approved by the Experimental Animal Ethics Committee of Shandong University (Approval number: 20072).

### Immunohistochemistry assay

Fresh tissue from tumor xenografts was fixed with 4% paraformaldehyde for 24 h, dehydrated, and embedded in paraffin and immunohistochemical analysis was conducted on paraffin-embedded tumor tissue sections(5 µm). According to the manufacture’s instruction, tablets were handled conventionally. Tumor tissue sections were dewaxed, boiled in microwave antigen retrieval technique for 15 min to repair antigen, immersed in 3%H_2_O_2_ for 30 min to block endogenous peroxidase, blocked with 3% goat serum for 30 min to block non-specific antigens. The primary antibodies (Table 2) were added to the sections and incubated overnight at 4 °C. After washing, the sections were added to the biotin-labeled goat anti-rabbit IgG polymer and horseradish enzyme-labeled streptomycin for 30 min respectively. Positive signals were determined with diaminobenzidine (DAB). The sections were stained with hematoxylin, sealed and analyzed. The results were scored by two pathologists with no knowledge of patient characteristics. The staining intensity was scored as negative (score = 0), weak (score = 1), medium (score = 2), or strong (score = 3). According to the percentage of stained-positive tumor cells (0%-5%, 6%–25%, 26%–50%, 51%-75%, and 76%–100%, respectively), the staining extent score was on a scale of 0–4. By multiplying the staining extent score by the intensity score, a score ranging from 0 to 12 was calculated.

**Table 2:** The information of antibodies used for western blot, immunohistochemistry, and flow cytometry

Antibodies	Reactivity	Dilution	Catalogue	Application
GAPDH	Human	1:1000	Cell Signal Technology, #5174	WB
PARP1	Human	1:1000	Abcam, #ab191217	WB; IHC
PD-L1	Human	1:200;2ug/ml	Abcam, #ab205921	WB; IHC
STING	Human	1:1000	Cell Signal Technology, #13,647	WB
p-STING_S366	Human	1:1000	Cell Signal Technology, #50,907	WB
TBK1	Human	1:1000	Cell Signal Technology, #3504	WB
p-TBK1_S172	Human	1:1000	Cell Signal Technology, #5483	WB
IRF3	Human	1:1000	Cell Signal Technology, #11,904	WB
p-IRF3_S396	Human	1:1000	Cell Signal Technology, #29,047	WB
CD8	Human	1:250	Abcam, ab93278	IHC
IFN-γ	Human	1:50	Novus, #JM10-10	IHC
CD3 and isotype	Human	5 μl of antibody per test	Elabscience, E-AB-F1001E	Flow cytometry
CD4 and isotype	Human	5 μl of antibody per test	Elabscience, E-AB-F1109I	Flow cytometry
CD8 and isotype	Human	5 μl of antibody per test	Elabscience, E-AB-F1110D	Flow cytometry
PD-L1 and isotype	Human	5 μl of antibody per test	Elabscience, E-AB-F1133E	Flow cytometry
IFN-γ and isotype	Human	5 μl of antibody per test	Thermo Fisher Scientific, 48–7319-41	Flow cytometry
CD3 and isotype	Mouse	5 μl of antibody per test	Elabscience, E-AB-F1013J	Flow cytometry
CD4 and isotype	Mouse	5 μl of antibody per test	Elabscience, E-AB-F1097C	Flow cytometry
CD8 and isotype	Mouse	5 μl of antibody per test	Elabscience, E-AB-F1104D	Flow cytometry
IFN-γ and isotype	Mouse	5 μl of antibody per test	Elabscience, E-AB-F1101E	Flow cytometry

### Western-blotting assay

After the treatment of different PARP inhibitors and Niraparib with concentrations of 0 nM, 500 nM, 1 μM, 5 μM, 10 μM, and 15 μM, proteins were collected after cell lysis. Total protein was separated and transferred to polyvinylidene fluoride membranes. Membranes were blocked and incubated with the primary antibody (Table 1) at 4 °C overnight. Samples were washed and incubated with secondary antibody at 37 °C for 1 h. Bands were detected using a chemiluminescent substrate (Thermo Fisher Scientific Inc. MA, USA) and then developed. The GAPDH band was served at control.

### CD8^+^T cell isolation and activation

Blood samples were collected from healthy donors, an equal volume of PBS was added. Ficoll lymphocyte separation medium (TBD Science, LTS1077, Tianjin, China) was used to isolate the peripheral blood mononuclear cells (PBMCs). After PBMC separation, CD8 immunomagnetic beads (20 μl/10^7^ cells) purchased from Miltenyi (Germany) were added and incubated at 4 °C for 15 min. The MS column was prewashed and used to separate CD8^+^T cells. Before the activation of T cells, cytokines (Human Recombinant IL-2, Peprotech) 20 ng/ml were added to ImmunoCult™-XF T Cell Expansion Medium (Stemcell Technologies, Canada) and mix thoroughly. To activate T cells, add 25 µl/ml of ImmunoCult™ Human CD3/CD28 T Cell Activator(Stemcell Technologies, Canada) to the cell suspension. Incubate cells at 37 °C and 5% CO_2_ for up to 3 days. After 3 days of activation, the cells were further cultured 7–10 days for further experiments.

### The co-culture assay of tumor cells and CD8^+^ T cells

Tumor cells were seeded in a 6-well plate with PBS, Niraparib, PD-L1 blockade, and the combination. Human peripheral blood mononuclear cells were isolated and activated and then cocultured with tumor cells at a 10:1 ratio.

### Flow cytometry analysis of PBMCs and cytokines

Blood samples were collected during treatment with Niraparib. For surface marker staining, 100 μl whole blood was stained with FITC-conjugated anti-human CD3, Percp-Cy5.5-conjugated anti-human CD4, and PE-conjugated anti-human CD8.

For intracellular cytokine staining, whole blood was stimulated with polyclonal cell activation mixture (2ul/ml cell culture) containing the phorbol ester-PMA (Phorbol 12-Myristate 13-Acetate), a calcium ionophore-Ionomycin and the protein transport inhibitor-Brefeldin A (Leukocyte Activation Cocktail, BD, Biosciences, #550583, San Jose, USA) for 4–6 h. The cells were first stained with surface markers for 30 min. Cells were fixed and permeabilized with BD Cytofix/Cytoperm (BD Bioscience, San Jose, USA) and then washed in FACS buffer (PBS with 5% FBS). Intracellular staining was performed using APC-conjugated anti-human IFN-γ.

A total of 80,000 events were collected per sample using flow cytometry (BD Bioscience, San Jose, USA). Flow cytometry data were analyzed using Flowjo software Version 10.0.

### Enzyme-linked immunosorbent assay (ELISA)

Cell culture supernatant with or without Niraparib (15 µM) from three times experiment was collected by centrifugation, and cytokine levels were measured according to the instructions of ELISA kits from Multisciences (Hangzhou, Zhejiang, China).

### Statistical analysis

GraphPad Prism software (version 8.0) and SPSS Statistics software (Version 24.0) were used to perform data analysis. Continuous and noncontinuous variables were analyzed by one-way ANOVA, Student’s t-test, and chi-square test, separately. The cutoff value of continuous data was calculated by the median. The Kaplan–Meier method with the log-rank test was used to calculate the risk factor related to the overall survival (OS). *P* < 0.05 was considered statistically significant.

## Results

### PARP inhibitors upregulate PD-L1 expression of ovarian cancer cells in vitro

**Fig. 1:**
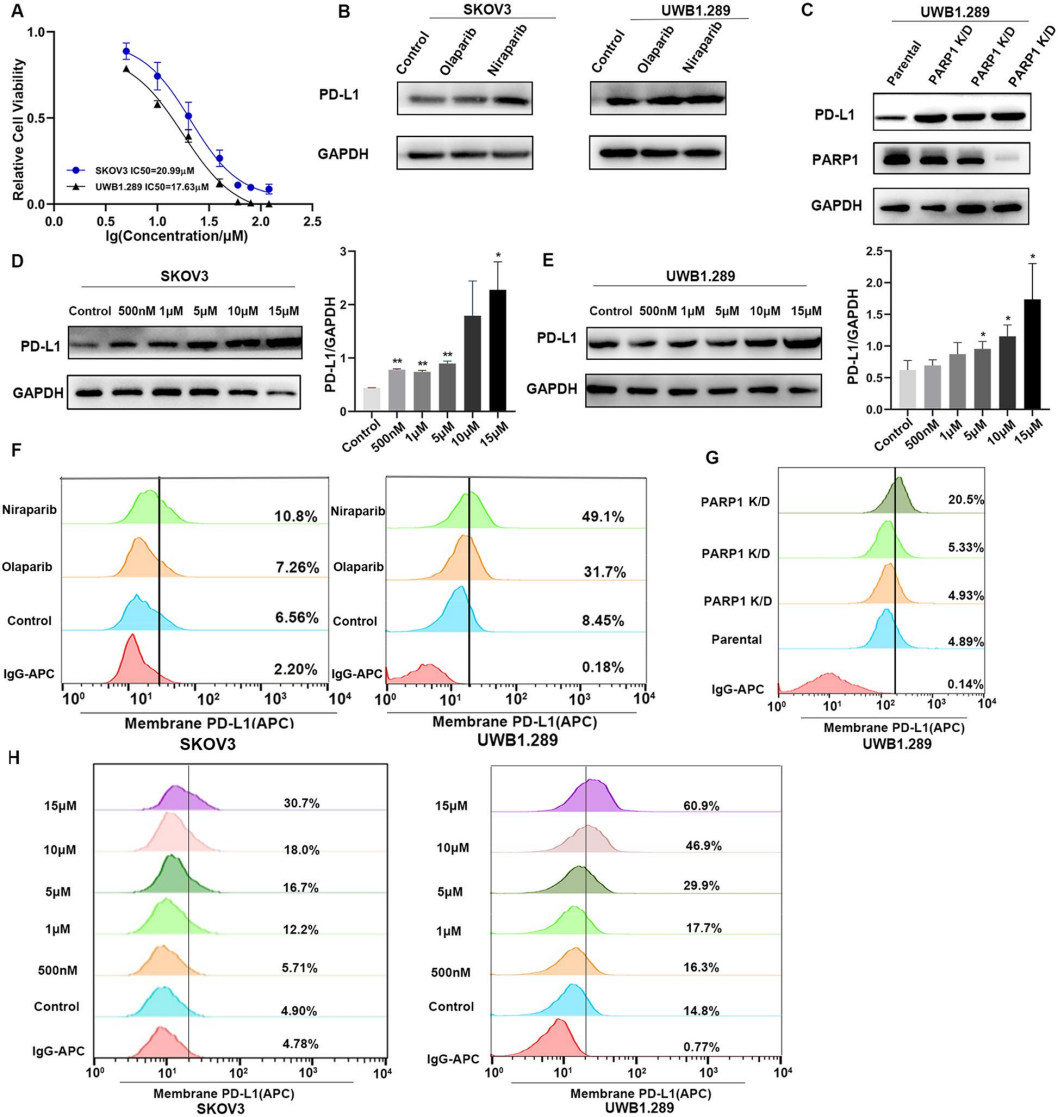
PARP inhibitors upregulate PD-L1 expression of ovarian cancer cells in vitro. **A** The IC_50_ of Niraparib in SKOV3 and UWB1.289 cells. **B** SKOV3 and UWB1.289 cells were treated with different PARP inhibitors for 48 h and the expression of PD-L1 protein was analyzed by western blotting. **C** SKOV3 and UWB1.289 cells were transfected with PARP1 siRNA. The PARP1 and PD-L1 expression were analyzed by western blotting. **D**, **E** SKOV3 and UWB1.289 cells were treated with different concentrations of Niraparib (0 nM, 500 nM, 1 μM, 5 μM, 10 μM, and 15 μM) and the expression of PD-L1 protein was analyzed by western blotting. **F** SKOV3 and UWB1.289 cells were treated with different PARP inhibitors for 48 h and the expression of PD-L1 on tumor surface was analyzed by flow cytometry. **G** UWB1.289 cells were transfected with PARP1 siRNA and the expression of PD-L1 on tumor surface was analyzed by flow cytometry. **H** Flow cytometry analysis of PD-L1 expression in two cells treated with different concentrations of Niraparib. All experiments were repeated three times. Data are presented as the mean ± SD. *P < 0.05, **P < 0.01

Increased PD-L1 expression in cancer cells has been shown to enhance PD-L1/PD-1 axis-mediated anticancer immunosuppression. To determine whether PARP inhibitors affect the PD-L1 protein expression, we detected the IC50 of different PARP inhibitors in two cell lines (Fig. 1A, Additional file 1: Figure S1A). Then we treated two types of ovarian cancer cells, SKOV3 and UWB1.289, with two different PARP inhibitors, Olaparib, Niraparib (10 μM) for 48 h. Cells treated with PARP inhibitors were collected and western blotting (WB) analysis was performed. PARP inhibitors upregulated the expression of PD-L1 protein in both cell lines, and Niraparib increased the expression of PD-L1 steadily (Fig. 1B, Additional file 1: Figure S1B). Therefore, we selected Niraparib as a representative agent for following up experiments. To explore whether PD-L1 elevation is specifically due to inhibition of PARP and is not an off-target effect of the inhibitor, we also transfected both cell lines with PARP siRNA and determined the expression of PD-L1 by WB and FACS (Fig. 1C, Additional file 1: Figure S1C, S1D). The results are consistent with the pharmacologic inhibition, and PD-L1 expression was increased in PARP knockdown cells compared with the parental control. Treatment with different concentrations of Niraparib (0 nM, 500 nM, 1 μM, 5 μM, 10 μM, and 15 μM) for 48 h upregulated PD-L1 expression in SKOV3 and UWB1.289 cells (Fig. 1D, E).

Previous studies showed that PD-L1 expressed on the cancer cell surface could exert immunosuppressive effects by binding to its receptor on activated T cells. We treated SKOV3 and UWB1.289 cells with different PARP inhibitors, transfected PARP1 siRNA, and treated with different concentrations of Niraparib to explore whether the cell surface PD-L1 levels increased after Niraparib treatment. Cell surface PD-L1 expression increased significantly after PARP inhibitor treatment (Fig. 1F, 1G, 1H, Additional file 1: Figure S1E). Together, these results suggest that PARP inhibitors can increase total and cell surface PD-L1 expression in ovarian cancer cells.

### Niraparib upregulates PD-L1 expression of ovarian cancer in vivo and has a synergistic effect with PD-L1 blockade

**Fig. 2:**
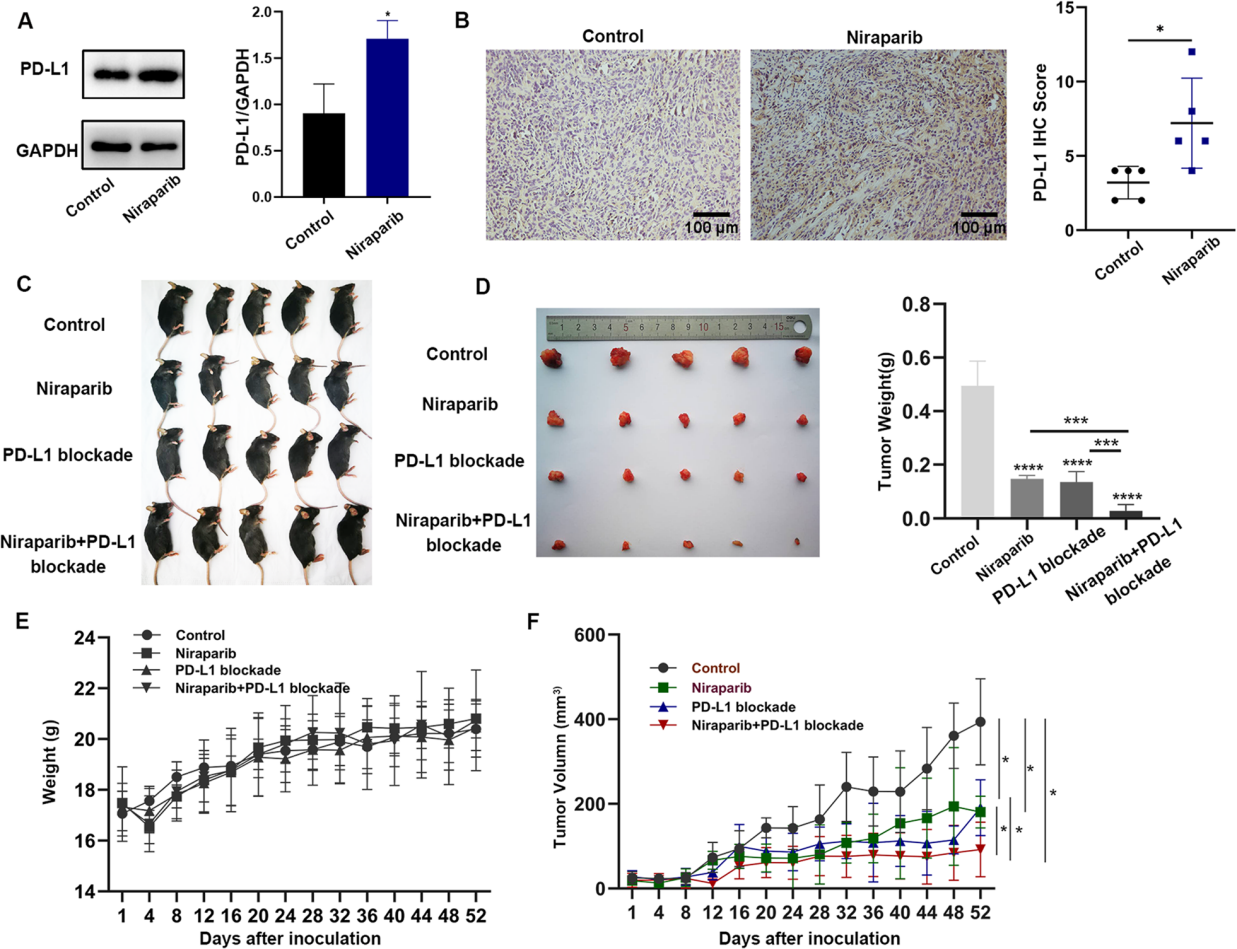
Niraparib upregulates PD-L1 expression of ovarian cancer cells in vivo and has a synergistic effect with PD-L1 blockade. **A** Western blot (WB) analysis of the PD-L1 expression in mouse tumor tissues from the control and Niraparib treatment (25 mg/kg, four times a week) group. **B** Representative images of immunohistochemistry (IHC) staining of PD-L1 in mouse ovarian cancer samples. Scale bar:100 μm. **C**, **D** Tumor volumes of four treatment groups with control (PBS, 100 μl), Niraparib (25 mg/kg), PD-L1 blockade (10 mg/kg, twice of the week), and Niraparib with PD-L1 blockade. **E** Weight curve of immunocompetent C57BL/6 mice treated with control, Niraparib, PD-L1 blockade, and the combination for 52 days. **F** Tumor growth curve of immunocompetent C57BL/6 models treated with control, Niraparib, PD-L1 blockade, and the combination for 52 days. Data are presented as the mean ± SD. *P < 0.05, **P < 0.01, ***P < 0.001

The above in vitro experiments indicate that Niraparib increases the expression of PD-L1 in ovarian cancer cells. Next, to explore whether PARP inhibitors could affect PD-L1 expression in mouse models, we inoculated ID8 cells into the right flank of C57BL/6 mice, and after tumors formed, the mice were treated with Niraparib (25 mg/kg, once a day, four times a week). Tumor tissues of xenografts were isolated and WB analysis was performed. PD-L1 expression was significantly higher in tumors treated with Niraparib than in those from the untreated mice (Fig. 2A). We also assessed PD-L1 expression by IHC staining of tumor tissues from mice. These results are consistent with those in vitro experiments (Fig. 2B). Therefore, Niraparib could increase PD-L1 expression in ovarian cancer both in vitro and in vivo. Based on these results, we investigated the effect of the combination of the PARP inhibitor Niraparib and PD-L1 blockade in mice (Fig. 2C). While a single agent could inhibit the growth of the tumors, the combination of Niraparib and PD-L1 blockade treatment did hamper tumor growth more effectively than the control treatment (Fig. 2D, F). Besides, there was no significant difference in the body weight curve of mice, indicating the combination of Niraparib and PD-L1 blockade has a little adverse effect (Fig. 2E). These data indicate that, while Niraparib can treat tumors effectively, activation of part of the immune-inhibitory pathway restricts the efficacy of the single agent, which can be overcome by combining with ICBs.

### Niraparib treatment alters the proportion and function of immune cells in vivo

**Fig. 3:**
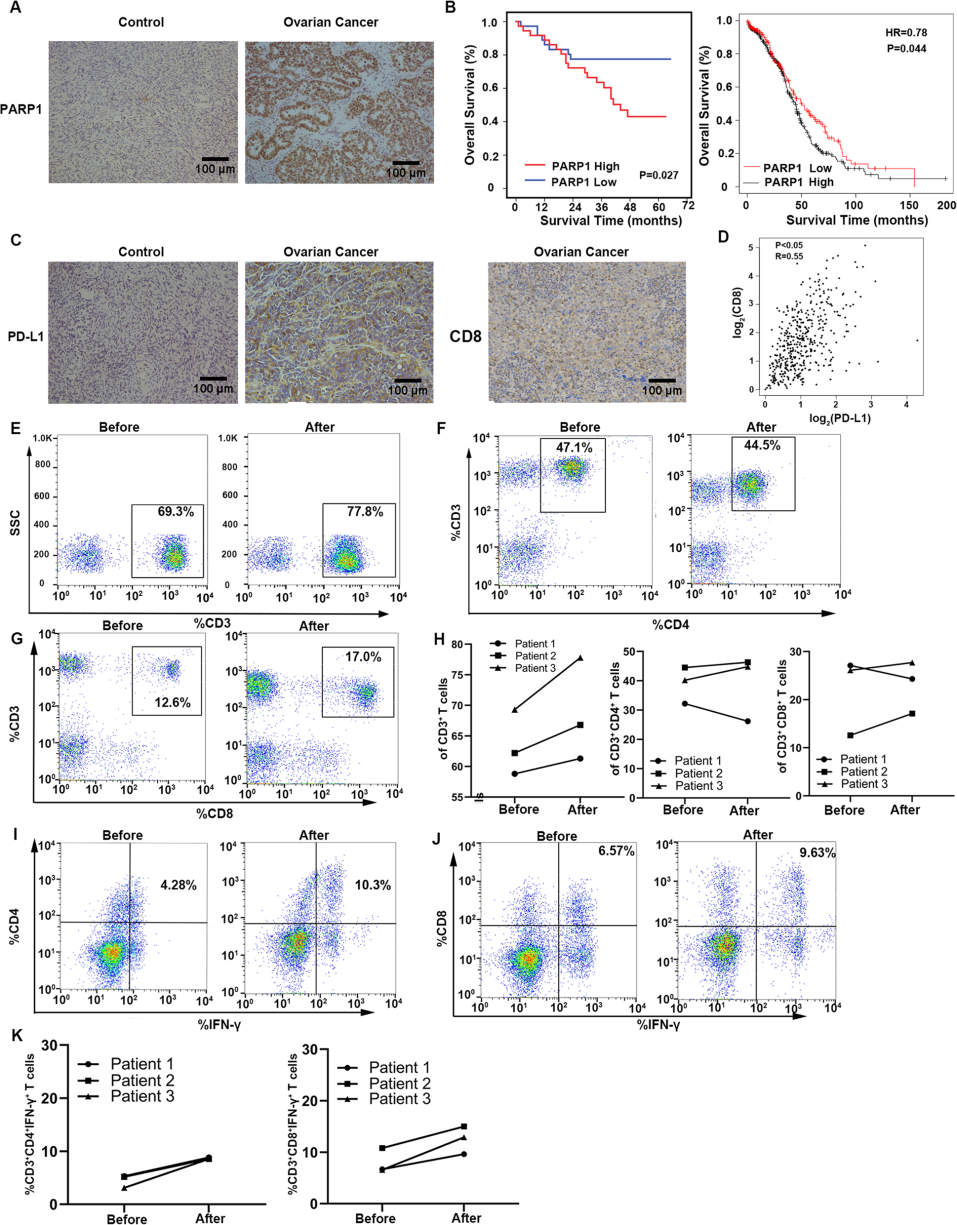
Niraparib treatment alters the proportion and function of tumor-associated lymphocytes in vivo. **A** Representative images of immunohistochemistry (IHC) staining for poly ADP-ribose polymerase (PARP1) in human normal ovarian tissues and ovarian cancer samples. Scale bar:100 μm. **B** Expression of PARP1 its relationship with clinical characteristics in ovarian cancer. **C** Representative images of IHC staining for programmed cell death ligand (PD-L1) and CD8 in human normal ovarian tissues and ovarian cancer samples. Scale bar:100 μm. **D** The correlation of CD8a and PD-L1 in GEPIA database. **E**-**K** Flow cytometry analysis of CD3^+^T cells, CD3^+^CD4^+^T cells, CD3^+^CD8^+^T cells, CD4^+^IFN-γ^+^ cells, and CD8^+^ IFN-γ^+^ cells of the Niraparib treatment patients. All experiments were repeated three times. Data are presented as the mean ± SD. *P < 0.05, **P < 0.01

PD-L1 binds with PD-1 and transmits inhibitory signals to the cells, which can induce apoptosis and incapacitation of CD8^+^ T cells. However, the expression and relationship between PD-L1 and CD8 on ovarian cancer is still lacking in sample studies and the correlation of PARP1, PD-L1 and CD8 remains unclear. Seventy-two patients with high-grade serous ovarian cancer were included in the retrospective analysis of our study to determine the relationship between PARP1, PD-L1, and CD8 (Table 3). IHC images showed the different expression of PARP1 in normal ovarian tissues and ovarian cancer tissues (Fig. 3A). The log-rank test and KMplotter analysis showed that ovarian cancer patients with increased PARP1 protein expression had poorer overall five-year survival (Fig. 3B). IHC images showed the expression of PD-L1 and CD8 in normal ovarian tissues and ovarian cancer tissues (Fig. 3C). And the result from GEPIA (Gene Expression Profiling Interactive Analysis) database revealed that PD-L1 had a positive correlation with CD8 (Fig. 3D). However, there was no direct correlation between PARP1, PD-L1, and CD8, considering the limited samples. To better elucidate the impact of Niraparib on immune cells, we collected peripheral blood of three patients receiving Niraparib treatment and compared the levels of T lymphocytes and cytokines. The clinical characteristics of the three patients were shown in Table 4. The results revealed that the proportion of CD4^+^ and CD8^+^ T lymphocytes decreased in one of the Niraparib-treated patients, while the proportion of CD3^+^, CD4^+^, and CD8^+^T lymphocytes increased in other two patients and cytokines secreted by T cells increased in three patients (Fig. 3E–K).

**Table 3:** Clinical characteristics between PARP1 low and high expression groups

Characteristic	Total (n = 72)	PARP1 expression	PARP1 expression	*P-*value
		Low	High	
Age (year)				0.238
< 55	36 (50.0)	21 (29.2)	15 (20.8)	
≥ 55	36 (50.0)	15 (20.8)	21 (29.2)	
FIGO stage (2014)				0.710
I and II	8 (11.1)	3 (4.2)	5 (6.9)	
III and IV	64 (88.9)	33 (45.8)	31 (43.1)	
Diameter (cm)				0.813
< 8	40 (55.6)	19 (26.4)	21 (29.2)	
≥ 8	32 (44.4)	17 (23.6)	15 (20.8)	
Ascitic fluid				0.312
Yes	23 (31.9)	9 (12.5)	14 (19.4)	
NO	49 (68.1)	27 (37.5)	22 (30.6)	
CA125 (U/ml)				0.115
< 35	4 (5.6)	0 (0.0)	4 (5.6)	
≥ 35	68 (94.4)	36 (50.0)	32 (44.4)	
PD-L1 expression				0.341
Low	41 (56.9)	23 (31.9)	18 (25.0)	
High	31 (43.1)	13 (18.1)	18 (25.0)	
CD8 expression (cancer tissue)				0.240
Low	38 (52.8)	17 (23.6)	21 (29.2)	
High	34 (47.2)	19 (26.4)	15 (20.8)	

Values are n (%)

FIGO, International Federation of Gynecology and Obstetrics; CA125, carbohydrate antigen; PARP1, poly-ADP-ribose polymerase 1; PD-L1, programmed death ligand 1

**Table 4:** The clinical characteristics of three patient with Niraparib treatment

	Age	Type	Bilateral ovaries	FIGO	CA125(Before)	CA125(After)	BRCA	Tumor metastasis	Surgery	Chemotherapy
Patient 1	45	HGSC	Yes	IIIC	6.96	7.2	Wild	No	Yes	Yes
Patient 2	58	HGSC	Yes	IV	13.2	12.3	Wild	No	Yes	Yes
Patient 3	60	HGSC	Yes	IV	7.55	11.1	Wild	No	Yes	Yes

### Niraparib treatment enhances the proportion and activation of T lymphocytes in the co-culture system

**Fig. 4:**
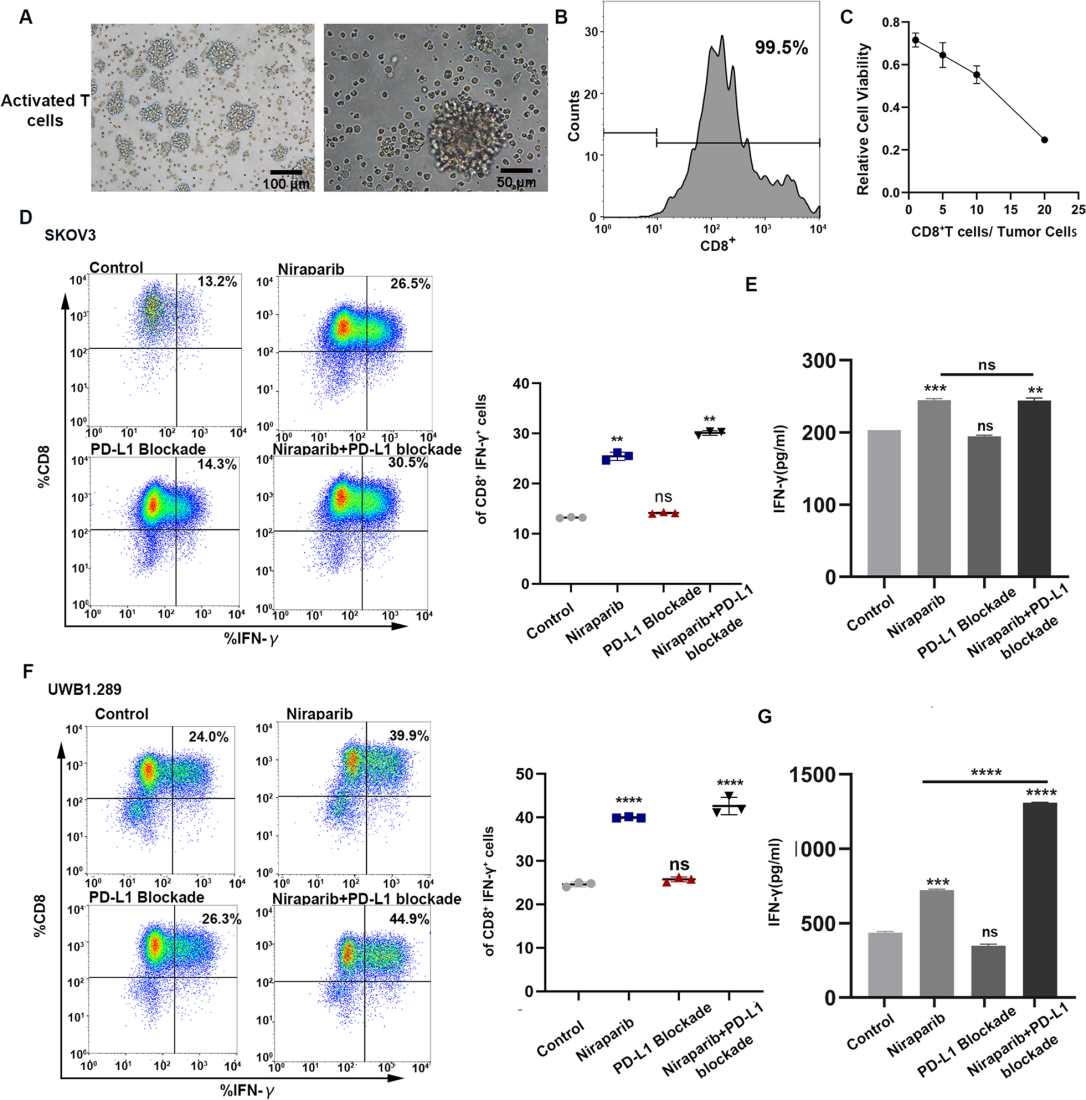
Niraparib treatment enhances the proportion and function of T lymphocytes in the co-culture system. **A** The morphological characteristics of activated T cells. Scale bar:100 μm. **B** The sorting efficiency of CD8^+^T cells. **C** The cytotoxicity of T cells to tumor cells in different ratios. **D**, **F** Immune profiling was analyzed by flow cytometry of the co-culture system with different treatments of control, Niraparib, programmed cell death ligand 1 (PD-L1) blockade, and the combination. **E**, **G** Enzyme-linked immunosorbent assay (ELISA) analysis of the cytokine from the co-culture system supernatant with different treatments. All experiments were repeated three times. Data are presented as the mean ± SD. *P < 0.05, **P < 0.01

The finding that Niraparib can elicit an anti-tumor immune response in patients prompted us to assess changes in immune cells and cytokines in the co-culture system. And the morphology of activated T cells was detected (Fig. 4A). We also determined the sorting efficiency of CD8 + T cells and the appropriate ratio of effective cells to targeted cells (Fig. 4B, C). We examined the effect of Niraparib, PD-L1 blockade, and the combination on the activation and function of T cells in vitro. Activated CD8^+^T cells isolated from peripheral blood of healthy donors were co-cultured with the UWB1.289 and SKOV3 tumor cells in a 10:1 ratio. We then treated the co-culture system with PBS, Niraparib, PD-L1 blockade and the combination for 48 h. After the co-culture, T cells were collected and stained with intracellular cytokine markers. The results from the co-culture system showed that the secretion of cytokines in the Niraparib-treated group was increased significantly compared to the control group and the combination enhanced the effect even more than the Niraparib group (Fig. 4D, F). The ELISA results from the co-culture supernatant were consistent with these data (Fig. 4E, G).

### Combination of Niraparib with PD-L1 blockade can enhance the proportion and function of T lymphocytes in vivo

**Fig. 5:**
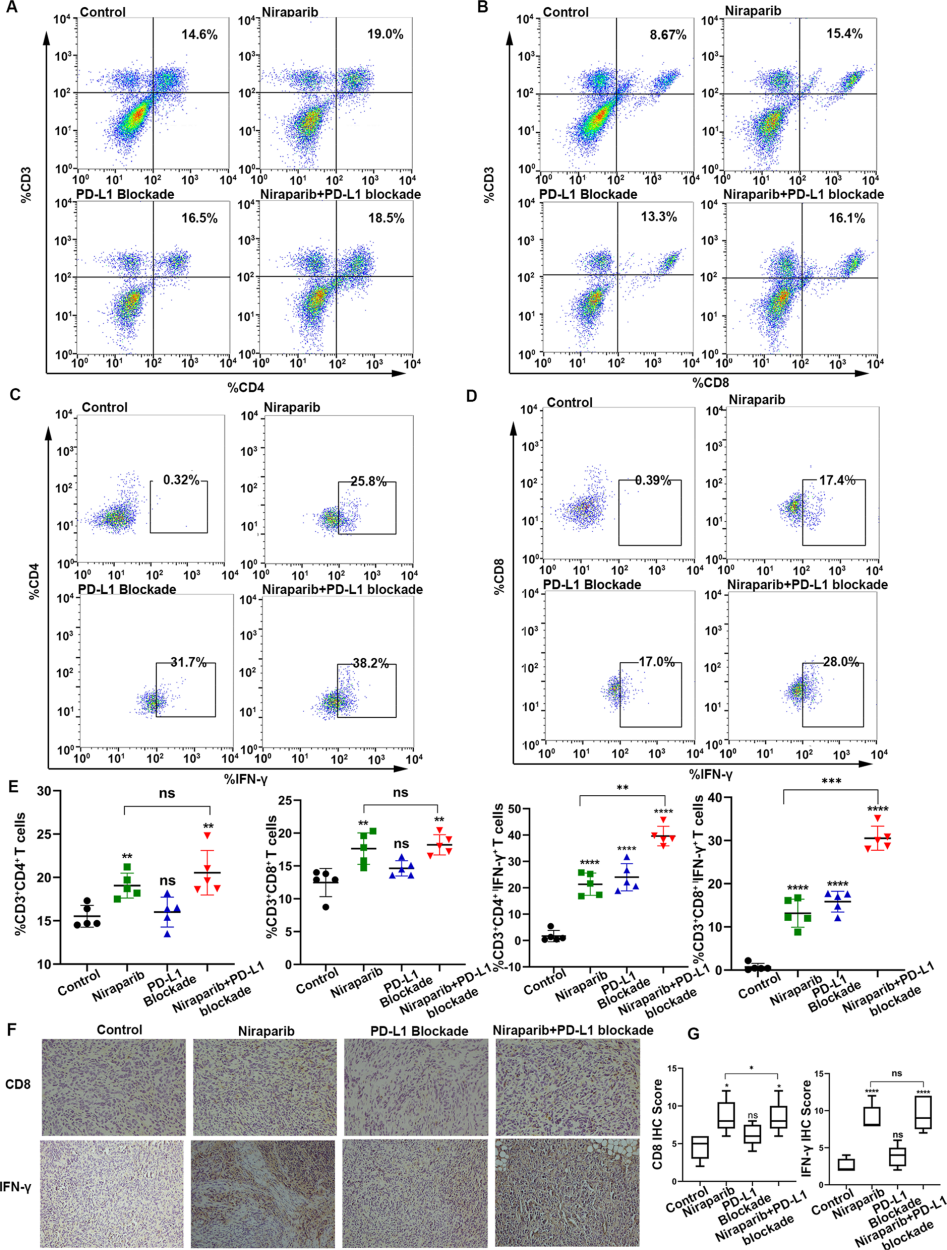
Combination of Niraparib with PD-L1 blockade can enhance the proportion and function of T lymphocytes in vivo. **A** Flow cytometry analysis of CD3^+^CD4^+^T lymphocytes from the peripheral blood of mice treated with control (PBS, 100 μl), Niraparib (25 mg/kg, four times a week), PD-L1 blockade (10 mg/kg, twice a week), and the combination. **B** Flow cytometry analysis of CD3^+^CD8^+^T lymphocytes from the peripheral blood of the mice from the four treatment groups. **C** Flow cytometry analysis of CD4^+^IFN-γ^+^T lymphocytes from the peripheral blood of the mice from the four treatment groups. **D** Flow cytometry analysis of CD8^+^IFN-γ^+^T lymphocytes from the peripheral blood of the mice from the four treatment groups. **E** Quantification of data of lymphocytes with four different treatment groups. **F** Representative images of immunohistochemistry (IHC) staining for CD8 and IFN-γ staining from resected tumors with different treatments at day 52. Scale bar: 50 μm. **G** Quantification of lymphocytes and IFN-γ with different treatment groups. All experiments were repeated three times. Data are presented as the mean ± SD. *P < 0.05, **P < 0.01

Based on previous observations, we found that Niraparib combined with PD-L1 blockade can hamper growth significantly and increase the proportion and function of T cells compared to single-drug treatment. To further confirm whether consistent changes were induced in the mouse models, we inoculated ID8 cells into the right flanks of C57BL/6 mice, and after the tumors formed, the mice were divided into the following treatment groups: control (PBS, 100 μl, intraperitoneal injection), Niraparib (25 mg/kg, oral administration, four times a week), PD-L1 blockade group (10 mg/kg, intraperitoneal injection, twice a week), and Niraparib (25 mg/kg) with PD-L1 blockade group (10 mg/kg). The mice were sacrificed at 52 days, and the changes in T cells and cytokine production were assessed in the four groups. Immune profiling by flow cytometry of the peripheral blood of mice showed CD4^+^ T cells and CD8^+^ T cells increased in the Niraparib group and combination group, but there was no significant difference in Niraparib group and combination group (Fig. 5A, B, E). There was a significant increase in the number of CD4^+^ IFN-γ^+^ cells and CD8^+^ IFN-γ^+^ cells in the Niraparib and PD-L1 blockade treatment groups, especially in the combination group (Fig. 5C–E).

We next investigated the local immune response upon treatment with Niraparib, PD-L1 blockade and the combination by IHC. Further analysis suggested that Niraparib treatment resulted in an increase of the CD8^+^T cells, along with the increased production of IFN-γ, and the combination group enhanced the effect more significantly than the single-agent treatment (Fig. 5F, G). Together, these data indicate that Niraparib elicits an intratumoral immune response, and the combination of Niraparib and PD-L1 blockade seems to work better than the individual treatments.

Thus, in summary, while the PARP inhibitor Niraparib alone can affect the proportion and function of T cells, the combination of Niraparib and PD-L1 blockade causes remarkable elevation of CD8^+^ cytotoxic T cells and IFN-γ compared with the single-agent group.

### The mechanism of Niraparib in upregulating the proportion and function of T cells

**Fig. 6:**
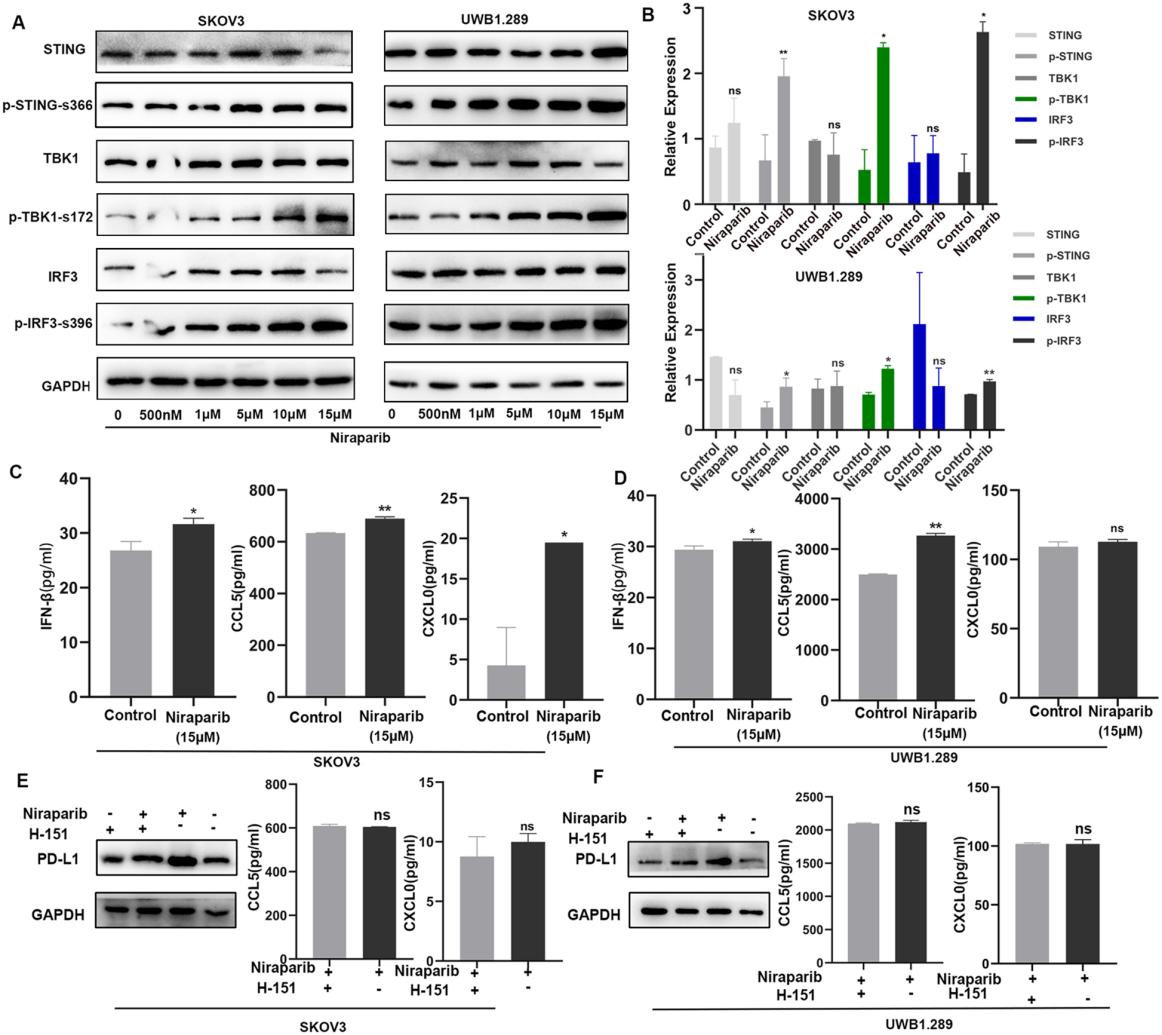
The mechanism of Niraparib in upregulating the proportion and function of T cells. **A** Western blot (WB) analysis of the STING pathway in SKOV3 and UWB1.289 cancer cells treated with different concentrations of Niraparib. **B** Quantification of data from the control and Niraparib treatment (15 µM) group. **C**, **D** Enzyme-linked immunosorbent assay (ELISA) analysis of the Interferon β (IFN-β) levels, chemokine (C–C motif) ligand 5 (CCL5) and, C-X-C motif chemokine 10 (CXCL10) from a co-culture system treated with control, Niraparib. **E**, **F** The cGAS/STING pathway was inhibited by H-151. The PD-L1, CCL5, and CXCL10 expression were analyzed by WB and ELISA after Niraparib treatment. All experiments were repeated three times. Data are presented as the mean ± SD. *P < 0.05, **P < 0.01

Based on the anti-tumor immune response we observed in a previous study, we hypothesized that the PARP inhibitors could induce immune regulation through the activation of the cGAS/STING innate immune pathway. To test whether the pathway was activated by the treatment with Niraparib, we treated the ovarian cancer cells with different concentrations of Niraparib (0 nM, 500 nM, 1 μM, 5 μM, 10 μM, and 15 μM). We observed that Niraparib treatment led to the activation of the STING pathway including p-TBK1-172 and p-IRF3-s396, in both ovarian cell lines (Fig. 6A). And the quantitative figure between the control group and Niraparib treatment (15 μM) group was shown (Fig. 6B). We examined the production of IFN-β, CXCL10, and CCL5 in the supernatants of the control and Niraparib treatment (15 μM) group, and the production of IFN-β, CXCL10, and CCL5 were increased significantly in the Niraparib treatment group, which explained the elevation of PD-L1 expression and the recruitment of CD8^+^T lymphocytes (Fig. 6C, D). Besides, we inhibited the activation of the pathway using STING inhibitor (H-151), the PD-L1, CCL5, and CXCL10 were not elevated in the Niraparib treatment group (Fig. 6E, F).

**Fig. 7:**
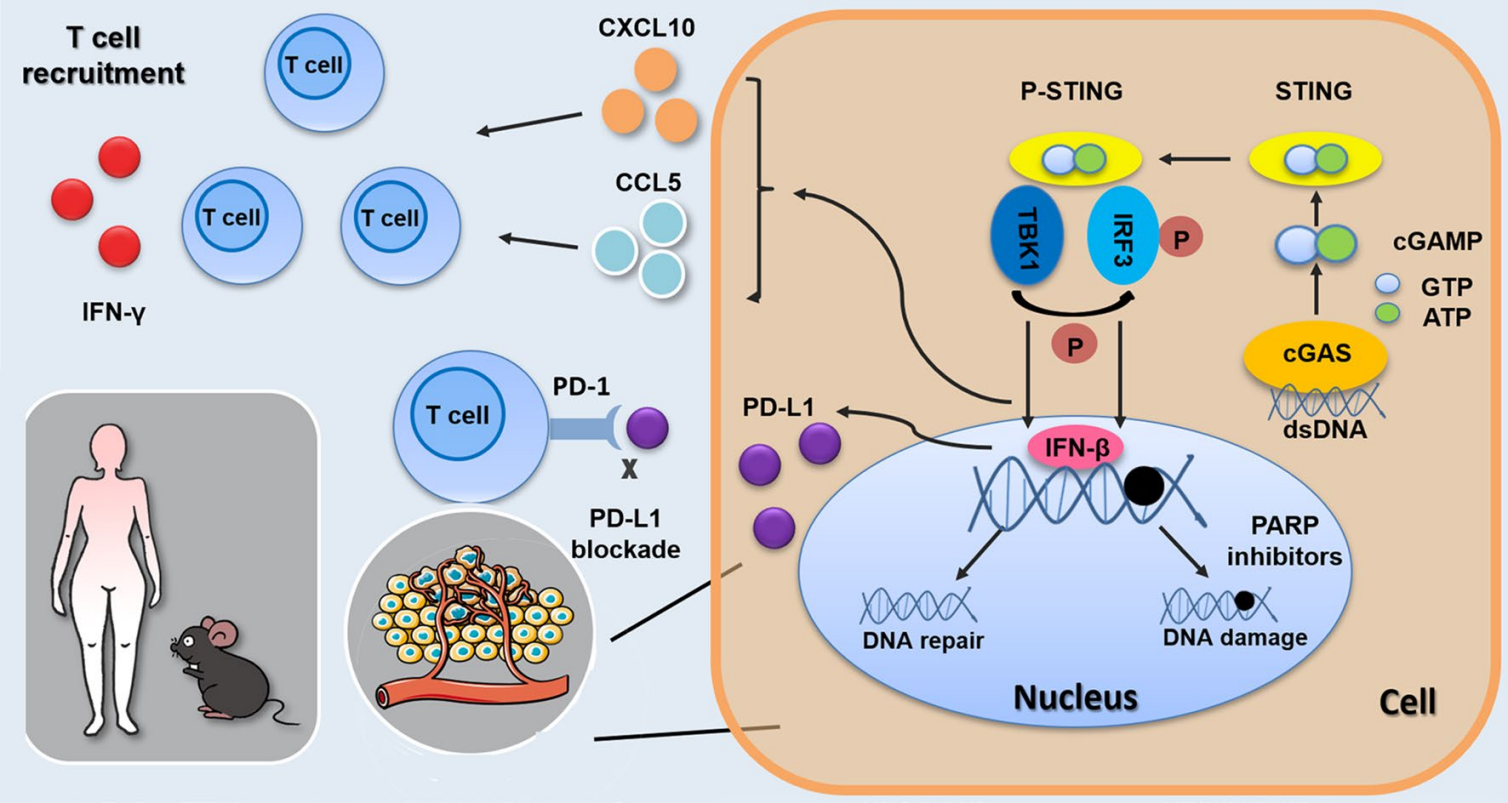
Model for STING pathway activation in response to PARP inhibitors in ovarian cancer

In conclusion, we observed that PARP inhibitor-induced DNA damage activated the STING pathway, which led to the upregulation of PD-L1 and recruitment of CD8^+^T lymphocytes via the induction of cytokines CXCL10 and CCL5 (Fig. 7). In summary, the PARP inhibitor Niraparib led to the recruitment of CD8^+^T cells and exerted an anti-tumor effect in our experiment, and PD-L1 blockade had a synergistic effect with Niraparib.

## Discussion

Ovarian cancer appears to be the most lethal malignancy in the female genital system. Two contributors to this phenomenon are asymptomatic at the early stage and chemotherapy resistance. The prognosis of high-grade ovarian cancer (HGOC) is poor, with a five-year survival of only 47.4%[12]. Therefore, there is an urgent need to find ways to improve the prognosis of patients. In recent years, significant progress has been made in understanding the underlying molecular biology of ovarian cancer and potential targets have been identified. PARP inhibitors and ICBs are both targeted therapeutic strategies under pre-clinical and clinical development for ovarian cancers and have been used in clinical treatment.

In the past several decades, remarkable breakthroughs have been made in exploring tumor immunosuppression. Multiple inhibitory ligands, especially PD-L1, are expressed on the tumor cell surface, which mediates the main cancer immune evasion pathway [13]. PD-L1 binds to its receptor PD-1 expressed on T cells, leading to the suppression of T cell proliferation and secretion while immune checkpoint inhibitors restore T cell function [14]. Most success has been achieved with ICBs, such as CTLA-4 and PD-1/PD-L1 blockade. Ipilimumab, nivolumab, and other immune checkpoint inhibitors have been approved by the FDA to treat multiple types of cancer, such as melanoma, lung, and bladder cancer [15, 16]. In the past few decades, despite great enthusiasm for immunotherapies, especially antibodies that targeted inhibitory molecules and achieved a significant survival improvement in melanoma and non-small cell lung cancer, few ovarian cancer patients benefited from ICBs (only 15% with nivolumab and 8% with the pembrolizumab) [17, 18]. Based on the limited response, current trials started focusing on the combination of ICBs with other targeted therapies, such as PARP inhibitors [19].

Preclinical studies have shown that the combination may have synergistic effects. PARP inhibitors treat tumors with DNA repair defects by inducing synthetic lethality [20]. Also, the cytotoxicity generated by PARP inhibitors can release damaged DNA marking the neoantigen and genomic instability, which would be a pivotal factor in determining tumor immunogenicity [21, 22]. Hence, PARP inhibitor-treated ovarian cancer appears to be more sensitive to ICBs, which may be a potential mechanism for combination therapy.

Only a few researchers have explored the possible tumor microenvironment change after PARP inhibitor treatment. Previous studies showed that Olaparib upregulated PD-L1 expression and impaired the function of T lymphocytes [23, 24]. However, Talazoparib has been shown to drive cytosolic DNA accumulation and STING activation in vitro and mouse models of cancer, leading to increased infiltration by immune cells and enhanced functionality of CD8^+^T and NK cells [25, 26]. The effect of Niraparib on PD-L1 and immune cells has not yet been elucidated. Here, we report a previously unmined role of Niraparib in regulating the anti-tumor immune response in ovarian cancer models. Niraparib enhances the anti-tumor immune response through T-cell-mediated effects. When combined with PD-L1 blockade, Niraparib has a significant anti-tumor effect, suggesting that these combinations may be clinically significant and benefit more patients.

There has been little focus on the mechanism of PARP inhibitors and the immune system. Previous reports have confirmed that DNA damage is associated with the activation of the anti-tumor immune response, including the STING pathway [27, 28]. Upon recognition of pathogenic or self-DNA, the pathway is activated and subsequently produces type I interferons [29]. The constant production of type I IFN initiates the innate immune system and enhances the infiltration of T lymphocytes and the secretion of cytokines [30, 31].

Previous reports have shown that DNA damage that arises from cytotoxic agents can activate the STING pathway, an innate immune pathway activated by cytoplasmic DNA. Here, we explored the efficacy of PD-L1 blockade combined with Niraparib and investigated the potential mechanism of the combination. We observed that Niraparib led to the activation of the cGAS/STING pathway, thus upregulating IFN-β, which is the direct mechanism of elevated PD-L1 expression. On the other hand, the pathway can activate IRF3 and significantly increase the expression of CCL5 and CXCL10, which leads to T cell recruitment and enhances the function of lymphocytes in ovarian tumors. Our data demonstrated that Niraparib could upregulate PD-L1 expression both in vitro* and *in vivo. However, the elevation of PD-L1 did not exert a suppressive effect on lymphocytes. Conversely, Niraparib influenced the composition and function of tumor-associated lymphocytes in peripheral blood, which was associated with a good prognosis and played a critical role in the surveillance of ovarian tumor development [32, 33]. However, the alteration of immune cells in long-term treatment and whether the changes can reflect the response to the treatment is uncertain and needs to be explored in a long-term follow-up.

In conclusion, our study demonstrated the remarkable efficacy of the combination of Niraparib and PD-L1 blockade, which provided a strong scientific rationale for the combination in the clinical settings. Besides, we verified the efficacy of the cGAS/STING pathway in the treatment of Niraparib, and it can be a new target for the treatment of tumors considering the recruitment of T cells. Further studies will be necessary to explore the effect of targeting the cGAS/STING pathway in ovarian cancers and the effect of PARP inhibitors or ICBs.

## Supplementary Information

**Additional file 1: Figure S1.** PARP inhibitors upregulate PD-L1 expression of ovarian cancer cells in vitro.** Table S1.** Description of clinical data in patients with HGSC.

## Data Availability

The data used and/or analysis during the current study are available from the corresponding author on reasonable request.

## Abbreviations

PARP: Poly (ADP) ribose polymerase
PD-L1: Programmed death 1 ligand
FACS: Fluorescence-activated cell sorting
WB: Western blot
IHC: Immunochemistry
cGAS: Cytosolic DNA sensor cyclic GMP-AMP synthetase
STING: Stimulator of interferon genes
TBK1: TANK binding kinase 1
IRF3: Interferon regulatory Factor 3
