# Supplementary material for: Niraparib exhibits a synergistic anti-tumor effect with PD-L1 blockade by inducing an immune response in ovarian cancer
Source: J Transl Med. 2021 Oct 7;19:415. doi: 10.1186/s12967-021-03073-0 (PMC8499579; doi:10.1186/s12967-021-03073-0)
Supplement: Supplementary file 1 — Additional file 1: Figure S1. PARP inhibitors upregulate PD-L1 expression of ovarian cancer cells in vitro. Table S1. Description of clinical data in patients with HGSC. [file 12967_2021_3073_MOESM1_ESM.docx]

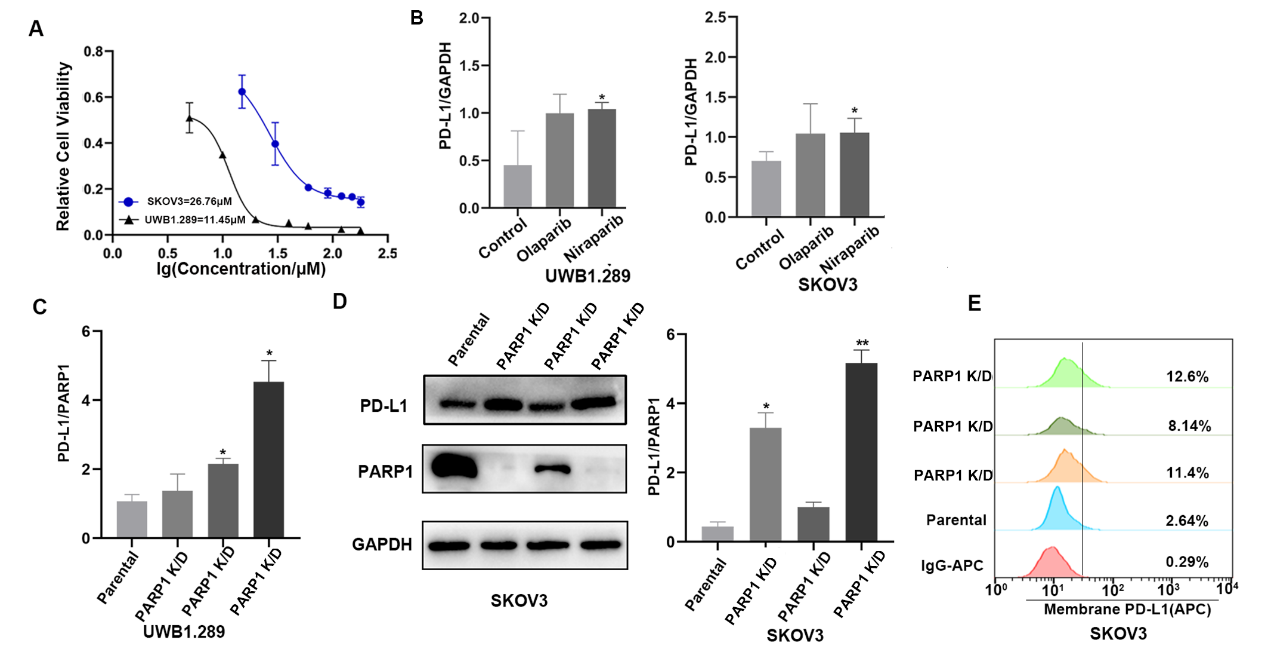


**Figure S1. PARP inhibitors upregulate PD-L1 expression of ovarian cancer cells *in vitro***

(A). The IC_50_ of Olaparib of SKOV3 and UWB1.289 cancer cells. (B). Western blot (WB) analysis of the total programmed cell death 1 ligand 1 (PD-L1) expression in SKOV3 and UWB1.289 ovarian cancer cells. (C). Quantification of PD-L1 expression in UWB1.289 cancer cells transfected with PARP1 siRNA. (D). PD-L1 expression in PARP1 knockdown (PARP1 K/D) and SKOV3 parental cells by immunoblotting and flow cytometry analysis

**Supplementary Table 1.** **Description of clinical data in patients with HGSC**

| **Characteristic** | **Median**  **(cutoff value)** | **Range of value** |
| --- | --- | --- |
| **Age (year)** | 55 | 36-77 |
| **Diameter (cm)** | 8.0 | 3.0-38.0 |
| **PARP1 expression** | 6.0 | 0.0-12.0 |
| **PD-L1 expression** | 8.0 | 0.0-12.0 |
| **CD8 expression (cancer tissue)** | 32.0 | 2.0-354.0 |

HGSC, high-grade serous ovarian cancer；PARP1, poly-ADP-ribose polymerase 1; PD-L1, programmed death ligand 1.
